# Analysis of Pectate Lyase Genes in Dickeya chrysanthemi Strain L11, Isolated from a Recreational Lake in Malaysia: a Draft Genome Sequence Perspective

**DOI:** 10.1128/genomeA.00145-15

**Published:** 2015-03-19

**Authors:** Kok-Gan Chan, Heng-Leong Kher, Chien-Yi Chang, Wai-Fong Yin, Kian-Hin Tan

**Affiliations:** a Division of Genetics and Molecular Biology, Institute of Biological Sciences, Faculty of Science, University of Malaya, Kuala Lumpur, Malaysia; b Interdisciplinary Computing and Complex BioSystems (ICOS) Research Group, School of Computing Science, Newcastle University, Newcastle upon Tyne, United Kingdom; c Centre for Bacterial Cell Biology, Medical School, Newcastle University, Newcastle upon Tyne, United Kingdom

## Abstract

Dickeya chrysanthemi is well known as a plant pathogen that caused major blackleg in the European potato industry in the 1990s. D. chrysanthemi strain L11 was discovered in a recreational lake in Malaysia. Here, we present its draft genome sequence.

## GENOME ANNOUNCEMENT

Dickeya chrysanthemi, formerly known as Pectobacterium chrysanthemi (1) and Erwinia chrysanthemi (2), is a typical phytopathogen that poses a great threat to crops, especially potatoes, which are a staple economic source in European countries. The Netherlands and Poland, in particular, have suffered great loss due to the manifestation of Dickeya spp. in potatoes (3, 4). It is interesting that D. chrysanthemi strain L11, which we present here, was found in a man-made recreational lake. This finding is essential for the study of its pathogenicity, as well as its genomic variation compared to members of the same species.

The genomic DNA of D. chrysanthemi L11 was extracted using a MasterPure DNA purification kit (Epicentre, Inc., Madison, WI, USA). DNA purity and concentration were analyzed, respectively, by Nanodrop spectrophotometer (Thermo Scientific, Waltham, MA, USA) and a Qubit version 2.0 fluorometer (Life Technologies, Carlsbad, CA, USA). Paired-end libraries were prepared using a Nextera DNA sample prep kit (Illumina, San Diego, CA, USA) prior to whole-genome sequencing using an Illumina MiSeq (Illumina, San Diego, CA, USA) sequencer. Assembly of the raw FASTQ sequences was performed by the use of CLC Genomics Workbench version 6.5.1 (CLC Bio, San Diego, CA, USA) (5). Low-quality sequence reads (limit 0.001), ambiguous nucleotides (*n* = 0), and sequence lengths of <50 nucleotides were eliminated for assembly. A total output of 4,149,494 paired-end reads with an average length of 200.2 bp was generated. The filtered reads were *de novo* assembled into 159 contigs. The resulting D. chrysanthemi L11 draft genome comprises 4,792,288 bases, with an average coverage of 64-fold and a G+C content of 54.2%. Gene prediction applying RAST (Rapid Annotations using Subsystem Technology) version 2.0 revealed a total number of 4,410 coding sequences and 71 RNAs.

From the annotated gene information, it was found that D. chrysanthemi L11 harbors multiple copies of pectate lyase genes in contigs 18, 22, 31, 44, 86, and 91, while pectin degradation protein KdgF is located in contig 71. Pectate lyase is an enzyme involved in the soft rot and maceration processes in plant tissues, particularly in the primary plant cell wall (6). The availability of the genome sequence of this isolate will facilitate research of D. chrysanthemi strain L11.

### Nucleotide sequence accession numbers.

This whole-genome shotgun project has been deposited at DDBJ/EMBL/GenBank under the accession number JSYH00000000. The version described in this paper is the first version, JSYH00000000.1.
